# The Anatomy of Sartorius Muscle and its Implications for Sarcoma Radiotherapy

**DOI:** 10.1080/13577140410001679194

**Published:** 2004-03

**Authors:** Neil G. Burnet, Tom Bennett-Britton, Andrew C. F. Hoole, Sarah J. Jefferies, Ian G. Parkin

**Affiliations:** 1 University of Cambridge Department of Oncology Oncology Centre (Box 193) Addenbrooke's Hospital Hills Road Cambridge CB2 2QQ UK; 2 Oncology Centre (Box 193) Addenbrooke's Hospital Hills Road Cambridge CB2 2QQ UK; 3 University of Cambridge Department of Anatomy Downing Site Downing Street Cambridge CB2 3DY UK; 4 Department of Medical Physics Addenbrooke's Hospital Hills Road Cambridge CB2 2QQ UK

## Abstract

*Purpose*: Controversy exists as to whether sartorius muscle is completely invested in fascia. If it is, then direct tumour involvement from soft tissue sarcoma of the anterior thigh would be unlikely and would justify omitting sartorius from the radiotherapy volume.

*Subjects and methods*: Eight thighs in six cadavers were examined in the dissecting room. Using a previous case, conformal radiotherapy plans were prepared to treat the anterior compartment of the thigh including and excluding sartorius. The corridor of unirradiated normal tissue was outlined separately.

*Results*: In all cases, sartorius was enclosed within a fascial sheath of its own. In four of the six cadavers, there was clear evidence of a fascial envelope surrounding sartorius, fused to the fascia lata and medial intermuscular septum. In two, sartorius was fully ensheathed in the upper half of the thigh; in the lower half the intermuscular septum became thin, and blended with the tendinous aponeurosis on the surface of vastus medialis in an example case. By excluding sartorius, the volume of the anterior compartment was reduced by 8%, but the volume of the unirradiated normal tissue corridor increased by 134%. With sartorius included, the unirradiated corridor became very small inferiorly, only 6% of the circumference of the whole leg, compared to 27% with sartorius excluded.

*Discussion*: The anatomy suggests that sartorius could be safely omitted from the clinical target volume of anterior compartment soft tissue sarcomas. This substantially increases the size of the unirradiated normal tissue corridor, expressed as a volume and a circumference, which could give a clinical advantage by reducing normal tissue complications.


*Sarcoma*, March 2004, *VOL. 8, NO. 1, 7–12*

Taylor & Francis healthsciences

ORIGINAL ARTICLE

### The anatomy of sartorius muscle and its implications for sarcoma radiotherapy

NEIL G. BURNET [1], TOM BENNETT-BRITTON[3], ANDREW C.F. HOOLE[4], SARAH J. JEFFERIES[2] & IAN G. PARKIN[3]

[1]*University of Cambridge Department of Oncology, Oncology Centre (Box 193), Addenbrooke's Hospital, Hills Road, Cambridge CB2 2QQ, UK;* [2]*Oncology Centre (Box 193), Addenbrooke's Hospital, Hills Road, Cambridge CB2 2QQ, UK;* [3]*University of Cambridge Department of Anatomy, Downing Site, Downing Street, Cambridge CB2 3DY, UK;* [4]*Department of Medical Physics, Addenbrooke's Hospital, Hills Road, Cambridge CB2 2QQ, UK*

**Abstract**

*Purpose*: Controversy exists as to whether sartorius muscle is completely invested in fascia. If it is, then direct tumour involvement from soft tissue sarcoma of the anterior thigh would be unlikely and would justify omitting sartorius from the radiotherapy volume.

*Subjects and methods*: Eight thighs in six cadavers were examined in the dissecting room. Using a previous case, conformal radiotherapy plans were prepared to treat the anterior compartment of the thigh including and excluding sartorius. The corridor of unirradiated normal tissue was outlined separately.

*Results*: In all cases, sartorius was enclosed within a fascial sheath of its own. In four of the six cadavers, there was clear evidence of a fascial envelope surrounding sartorius, fused to the fascia lata and medial intermuscular septum. In two, sartorius was fully ensheathed in the upper half of the thigh; in the lower half the intermuscular septum became thin, and blended with the tendinous aponeurosis on the surface of vastus medialis in an example case. By excluding sartorius, the volume of the anterior compartment was reduced by 8%, but the volume of the unirradiated normal tissue corridor increased by 134%. With sartorius included, the unirradiated corridor became very small inferiorly, only 6% of the circumference of the whole leg, compared to 27% with sartorius excluded.

*Discussion*: The anatomy suggests that sartorius could be safely omitted from the clinical target volume of anterior compartment soft tissue sarcomas. This substantially increases the size of the unirradiated normal tissue corridor, expressed as a volume and a circumference, which could give a clinical advantage by reducing normal tissue complications.

**Key words:** *radiotherapy, sartorius muscle*

### Introduction

When considering post-operative radiotherapy for soft tissue sarcoma of the limbs, it is conventional practice to irradiate the whole of the transverse cross-section of the affected anatomical compartment. In the specific case of sarcomas arising in the anterior compartment of the thigh, sartorius muscle is important because it slopes across the thigh. As it runs distally in the thigh, from its origin immediately below the anterior superior iliac spine to its insertion on the upper, medial surface of the tibia, so its position becomes progressively more medial and posterior. Thus, in the lower half of the thigh medial to the femur, it determines the posterior limit of the anterior compartment. Inclusion of this muscle in the radiotherapy target volume for the anterior compartment of the thigh leads to a larger volume treated, with a substantially reduced corridor of normal tissue remaining unirradiated, than if it could be excluded.

On both CT and MRI, it is clear that a fat plane normally exists around sartorius, separating it from the other muscles in the anterior compartment. We therefore hypothesised that sartorius might be separated from the quadriceps muscles by a more definite tissue plane than between other muscles in the same group. If a fascial membrane surrounds the muscle then direct tumour involvement would be most unlikely and would justify omission of the muscle from the radiotherapy clinical target volume (CTV).

In the anatomy literature controversy exists as to whether sartorius is completely invested in fascia, forming a separate compartment, or not. We investigated the morbid anatomy of the muscle to address this question.

Correspondence to: Dr Neil Burnet, University of Cambridge Department of Oncology, Oncology Centre (Box 193), Addenbrooke's Hospital, Hills Road, Cambridge CB2 2QQ, UK. Tel.: +44-1223-336800; Fax: +44-1223-763120; E-mail: ngb21@cam.ac.uk

1357-714X print/1369–1643 © 2003 Taylor & Francis Ltd
DOI: 10.1080/13577140410001679194

### Subjects and methods

### *Anatomical investigation*

The anterior compartments of eight thighs in six cadavers, which were available for first year pre-clinical anatomy dissection, were examined. The cadavers form an important element of anatomy teaching, so the dissection had to be carried out by the pre-clinical students themselves, under the supervision of the teaching staff. The students were made aware of our interest in the muscle sartorius and its fascial surrounds, but the focus of the anatomy teaching for the dissection was the major structures in the thigh, notably the muscles, vessels and nerves. The students were asked to pause as they removed the deep investing fascia of the thigh, to allow inspection of the fascial surrounds of sartorius, and as many dissections as possible were observed. However, since the purpose of the dissections was anatomy teaching, with a strict timetable to be adhered to, there was time to study only a few cadavers.

There were clear differences in the quality of tissues between cadavers. Some were fatter than others and there may be an optimum amount of body fat to preserve fat planes between muscles and around the fascia. In some cases, the tissues were harder than others, and in these cases it was more difficult to expose the muscles without destroying the surrounding fascia.

The relationship of sartorius to the fascia around it was observed and recorded on a proforma sheet. In particular, the dispositions of the medial intermuscular septum and the femoral sheath (the fascial envelope surrounding the femoral vessels) were examined. The upper and lower halves of the thigh were considered separately, to reflect any possible difference due to the change in compartment of the femoral vessels, passing from anterior to posterior through the subsartorial canal and adductor hiatus.

### *Radiotherapy planning*

In order to illustrate the potential impact of omitting sartorius from the anterior muscle compartment for radical radiotherapy, the radiotherapy planning CT and treatment plan for a previous case of soft tissue sarcoma were recalled. The anterior compartment was considered to be the CTV. The length of the CTV was set to be the same as the original plan. By specifying the anterior compartment as a Region of Interest, its volume is calculated by the treatment planning system and shown in the plan parameters. The anterior compartment was then outlined excluding sartorius muscle, and the new volume calculated. Full conformal radiotherapy plans were prepared, with sartorius included or excluded from the anterior compartment. The unirradiated normal tissue corridor was then outlined on each CT slice as a separate volume. This corridor was defined as the tissue in the thigh outside the projections of the treatment beams or conformal blocks, which should therefore receive less than 50% of the prescribed dose.

The size of the unirradiated corridor was described in two ways: firstly, the volume of the corridor was calculated; secondly, on each axial CT slice, the length of the skin surface in the unirradiated corridor (i.e., outside the projections of the beams or blocks) was measured and expressed as a proportion of the circumference of the whole thigh. Planning was done using our in-house Addenbrooke's radiotherapy planning system (ARPS). A specific additional tool was written into ARPS in order to measure the circumference of the thigh and normal tissue corridor.

For completeness, the planning target volume (PTV) was also measured. This is prepared by expanding the CTV using an isotropic growing algorithm to apply a 1-cm margin all round. This extends into the subcutaneous fat, and in parts may lie outside the skin. This volume may therefore have less relevance in assessing volume differences, but does reduce the corridor of unirradiated normal tissue.

### Results

### *The anatomy of the sartorius fascia*

In the cases where both thighs were examined, the same findings were noted in both. In four of the six cadavers, there was clear evidence of a fascial envelope surrounding sartorius, forming a continuous sheath (Table 1). The sheath was fused to, and indistinguishable from, the deep investing fascia and the medial intermuscular septum.

In the other two cadavers, sartorius was ensheathed in the upper half of the thigh. In the lower half, however, the fascial layer lying postero-medially (the intermuscular septum) became thinned, and appeared to blend with the tendinous aponeurosis on the surface of vastus medialis (Table 1).

**Table 1.** *Relationship of fascia to sartorius in eight legs from six cadavers*

| Cadaver | | Sartorius enclosed by fascia | |
|---|---|---|---|
| | | Thigh, upper half | Thigh, lower half |
| 1 | (R) | ✓ | ✓ |
| 2 | (L) | ✓ | ✓ |
| | (R) | ✓ | ✓ |
| 3 | (L) | ✓ | ✓ |
| | (R) | ✓ | ✓ |
| 4 | (L) | ✓ | ✓ |
| 5 | (L) | ✓ | ✓[a] |
| 6 | (R) | ✓ | ✓[a] |

[a]In these two cases, the fascial envelope was rather thin on the deep surface of the muscle distally, and was not distinguishable from the tendinous aponeurosis on the surface of vastus medialis.

The major difference in these two cases, compared to the other four, was the thickness of this fascial layer.

In all the cases, there was clear evidence that a layer of deep fascia forming the intermuscular septum lay postero-medial to the muscle, thus dividing sartorius from the posterior compartment. Likewise, in all the cases, the thickness of the fascia of the femoral sheath was notable, being clearly evident as a thick fascial layer deep to sartorius in the upper part of the thigh above the adductor canal, and thicker than the remainder of the sartorius sheath itself. In the lower half of the thigh, deep investing fascia disappears with the vessels, as they pass into the posterior compartment. This fascial layer would be unlikely to be breached by tumour beneath it.

Figure 1 shows a photograph of the sartorius muscle being retracted to reveal a fascial layer deep to it. The cut surface of the fascial envelope, essentially the edge of the deep investing fascia, is seen towards the top of the photograph. This photograph was taken in the mid-thigh level, and represents a typical example of those cases in which the fascia was present deep to sartorius for its entire length.

*Compartment volumes for planning radiotherapy*

Figure 2a shows a CT cross-section of the distal half of the thigh, from the planning scan used to calculate the anterior compartment volumes. Figure 2b shows the anatomical compartments and sartorius, drawn from the CT. These illustrate how far postero-medially the anterior compartment is extended by inclusion of the sartorius.

Figure 3 illustrates the radiotherapy plan, in diagrammatic form, for this patient illustrating how far posteriorly the high dose radiotherapy volume extends. This plan takes into account the twist of the anterior compartment along the length of the thigh, as well as the position of sartorius itself. Figure 3b shows the plan if sartorius is excluded from the CTV.

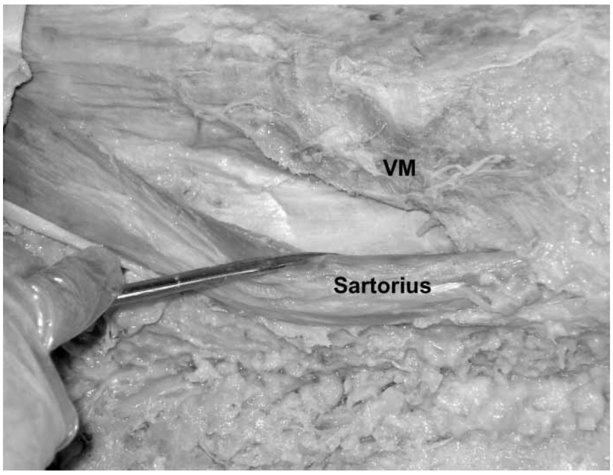

**Fig. 1.** *Close up view of the lower part of the left thigh, distal to the adductor canal. For orientation, proximal is to the left, distal to the right, anterior at the top. Sartorius, being retracted, is seen from the medial side. Anterior to this (above, in the picture) is the vastus medialis (VM). On the posterior edge of VM, the ragged cut edge of the fascial sheath surrounding sartorius can be seen. Deep to sartorius, visualised with the retraction, is further thick fascia, which is connected beneath sartorius to the medial intermuscular septum. Below this fascia in turn lies the somewhat aponeurotic surface of vastus intermedius.*

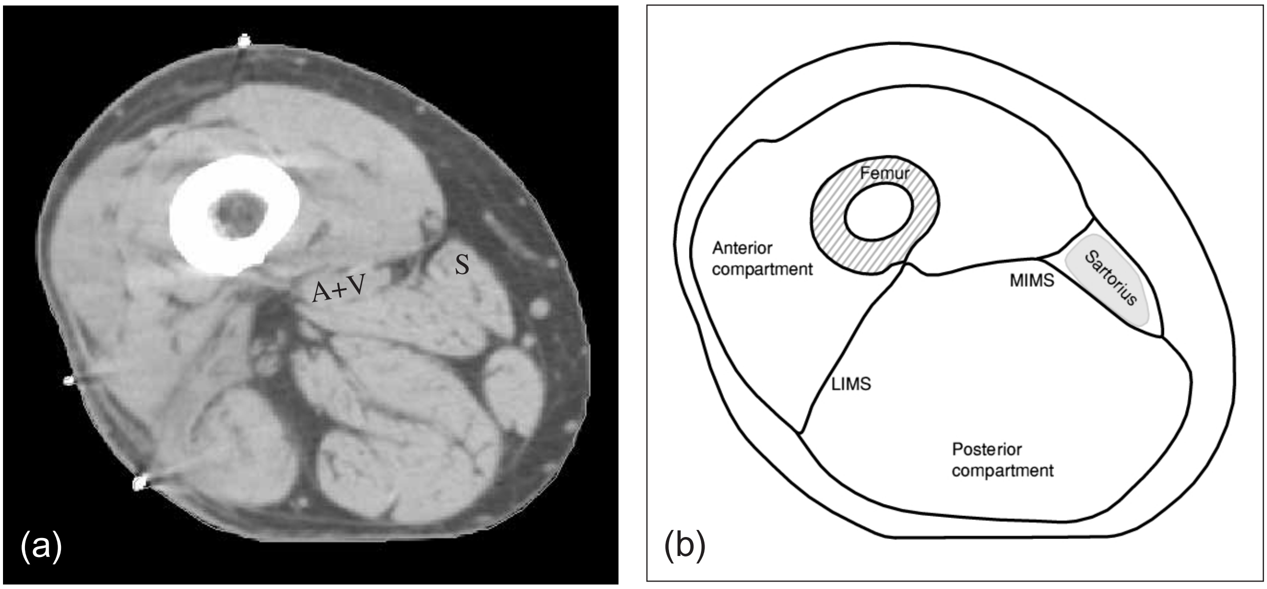

**Fig. 2.** *(a) Radiotherapy planning scan through the lower thigh, below the adductor canal. The main muscle groups can be seen, and sartorius is marked 'S'. The femoral vessels lie immediately posterior to the intermuscular septum within the posterior compartment and are marked 'A+V'. (b) Schematic drawing of the thigh, taken from the CT. Sartorius with its surrounding fascia is labelled. The medial and lateral intermuscular septa are also shown (MIMS & LIMS): they attach centrally to the femur along the linea aspera, and peripherally to the deep investing fascia of the thigh, the fascia lata.*

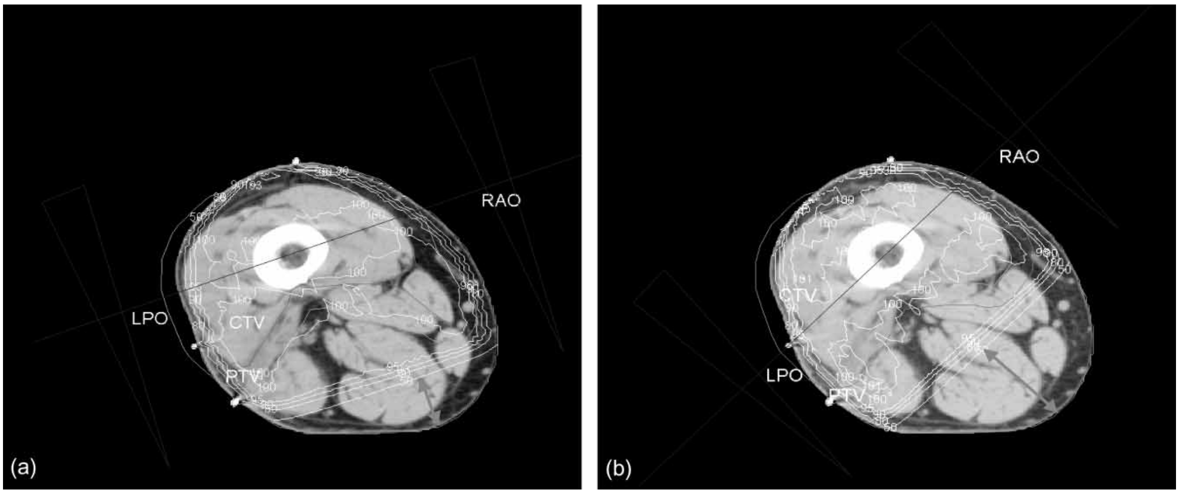

**Fig. 3.** *(a) Radiotherapy plan at the level shown in Fig. 2, 5.5 cm distal to the central axis of the radiotherapy plan, to treat the whole anterior compartment including sartorius. Note the width of the corridor of tissue which receives less than 50% of the dose, indicated by the double arrow. At this level the corridor is 27% of the circumference of the thigh, though it falls to only 6%, 7 cm below this, at the distal edge of the PTV. (b) Radiotherapy plan comparable to (a) but with sartorius excluded from the anterior compartment. The unirradiated corridor (arrowed) is somewhat larger, occupying 35% of the circumference of the thigh, and it never falls below 27%.*

**Table 2.** *Volume of the anterior compartment and unirradiated corridor, depending on whether sartorius is included or excluded*

| | Volume (ml) | | |
|---|---|---|---|
| | Anterior compartment | PTV[a] | Unirradiated corridor[b] |
| Sartorius included in anterior compartment | 1514 | 2311 | 523 |
| Sartorius excluded from anterior compartment | 1391 | 2081 | 1226 |
| After excluding sartorius | −8% | −10% | +134% |

[a]PTV, planning target volume.
[b]The unirradiated corridor is defined as the volume of normal tissue in the thigh outside the projections of the treatment beams or blocks, therefore receiving less than 50% of the prescribed dose.

**Table 3.** *The size of the unirradiated corridor, depending on whether sartorius is included or excluded, expressed as the length of the skin surface of the corridor in axial CT sections, and as a proportion of the circumference of the thigh*

| | Circumference of unirradiated corridor | | | | | |
|---|---|---|---|---|---|---|
| | Length of skin surface (cm) | | | Percentage of circumference of whole thigh | | |
| | Max | Mean | Min | Max | Mean | Min |
| Sartorius included in anterior compartment | 29 cm | 13 cm | 2.5 cm | 44% | 25% | 6% |
| Sartorius excluded from anterior compartment | 34 cm | 18 cm | 10 cm | 51% | 36% | 27% |
| Percentage change after excluding sartorius | +17% | +39% | +312% | | | |

The volume of the anterior compartment in the radiotherapy plan, depending on whether sartorius is included or excluded, is shown in Table 2. Although the anterior compartment volume is reduced by only 8% by excluding sartorius, the volume of the unirradiated normal tissue corridor is increased very substantially, from 523 to 1226 ml, an increase of 134%.

The size of the unirradiated corridor was also expressed as the proportion of the circumference of the thigh outside the projections of the beams or blocks (Table 3). For the plan including sartorius, the mean size of the unirradiated corridor was 25% of the circumference of the thigh. This became much smaller inferiorly, only 6% of the circumference. These figures contrast with a mean of 36% and a minimum of 27% of the circumference of the thigh, for the plan excluding sartorius. The difference in the size of the unirradiated corridor could translate into a clinical advantage in reducing normal tissue complications.

### Discussion

The purpose of this study was to examine the anatomy of the thigh to establish whether sartorius is fully surrounded by a fascial envelope which could protect it from invasion by soft tissue sarcomas arising nearby. If sartorius is protected, this would allow its exclusion from the anterior compartment for adjuvant radiotherapy planning, with consequent increase in the size of the corridor of unirradiated normal tissue. This in turn should improve the normal tissue outcome for patients treated for soft tissue sarcoma of the anterior thigh.

### *Anatomy of sartorius and its surrounding fascia*

Sartorius is a long strap muscle that originates from the anterior superior iliac spine, and inserts on the upper medial surface of the tibia. It crosses the anterior thigh obliquely, its medial edge forming the lateral border of the femoral triangle. It takes its innervation from a branch of the anterior division of the femoral nerve, and draws the lower limb into a tailor's sitting position, by flexing, laterally rotating, and abducting the hip, and flexing the knee. Sartorius is considered to lie in the anterior compartment of the thigh, whose muscles are all supplied by the femoral nerve.

The deep investing fascia which envelopes the muscles of the thigh is known as the fascia lata. It splits into two distinct layers at several locations in the thigh, either to enclose muscles such as gluteus maximus and tensor fascia lata, or to create openings such as the saphenous opening for the great saphenous vein. The fascia lata also joins to the medial intermuscular septum, which lies between the vastus medialis muscle in the anterior compartment, and the adductor longus and adductor magnus in the posterior compartment. This septum continues deeply to attach to the medial lip of the linea aspera of the femur, and its medial supracondylar ridge.[1,2] It might be expected that if sartorius is surrounded by its own fascial layer, this would facilitate movement of this long strap muscle over the underlying quadriceps.

The anatomical literature is divided as to whether sartorius is simply divided from the posterior compartment by the medial intermuscular septum, remaining in contact with the quadriceps muscles, or is completely invested by deep fascia in its own fascial compartment, formed by the medial intermuscular septum posteriorly and a separate layer passing from the septum to the fascia lata anteriorly. For example, Draves[3] states that the fascia lata does not completely surround and enclose sartorius; instead the deep layer continues to pass deeply, forming the medial intermuscular septum. This is contradicted by Wood Jones,[4] who describes and illustrates the sartorius sheath fully enclosing the muscle, and by Woodbourne and Burkel,[5] who suggest that the medial intermuscular septum splits to enclose sartorius. Many anatomy textbooks do not mention explicitly whether sartorius is completely enclosed by deep fascia or not, and diagrams in two recent textbooks[6,7] label the medial intermuscular septum as passing anteriorly, or posteriorly to sartorius, respectively.

Since this detail may alter clinical treatment, it was important for this to be clarified. Our investigation, though limited to eight thighs in six individuals, demonstrated that the intermuscular septum does lie posterior to sartorius, and that sartorius is enclosed within a fascial sheath of its own. In all eight thighs, there was evidence of a fascial envelope around sartorius in the upper thigh. Posteriorly, this is reinforced by the thick aponeurotic roof of the subsartorial canal,[4] which would protect sartorius, above the adductor canal, from invasion by a tumour sited deep and posterior to it. In the lower half of the thigh, in four out of six individuals, there was clear evidence that this envelope continued distally. In the lower half of the thigh in two subjects, the thickened subsartorial fascia appeared to merge with an aponeurotic layer on the surface of vastus medialis. It is less definite that this layer would prevent invasion of tumour although qualitatively this layer appeared to be as thick as the intermuscular septum, suggesting that it would be as effective at resisting tumour invasion as the septum. Since it is rare for soft tissue sarcomas to penetrate intermuscular septa, or the fascia lata, protection of sartorius should be as good as protection between the compartments.

### *Anatomy of sartorius and radiotherapy planning issues*

The anatomy of sartorius muscle is especially relevant for tumours of the anterior compartment because, as it passes down the thigh, it changes position from anterior to medial to postero-medial. In the lower half of the thigh, sartorius is the most posterior part of the anterior compartment medially. Thus, sartorius defines the posterior extent of the radiotherapy target volume medially, if it is included within the anterior compartment. The angle of the intermuscular septum in the thigh changes when moving from proximal to distal, typically introducing an internal rotation towards the distal end of the thigh. Sartorius contributes to this since it forms the postero-medial border of the anterior compartment distally. This twist in the position of the anterior compartment leads to an increase in the normal tissue treated in the posterior compartment, if the anterior compartment is fully encompassed. This accounts for the relatively large amount of posterior compartment tissue treated to the full prescription dose, shown in Fig. 3, and for the smaller than expected corridor of unirradiated normal tissue.

If it were possible to omit sartorius, then target volumes would be reduced and, as a consequence, the corridor of normal tissue left unirradiated would be increased substantially (Tables 2 and 3). It is an important principle that reducing the volume of normal tissue irradiated unnecessarily should be of benefit to the patient.[8] In the specific context of radiotherapy to the limbs, the functional outcome is related to radiotherapy volume and the size of the unirradiated corridor.[9–12] It has been recommended that this corridor should be at least one-third of the circumference of the limb.[13] In our case, Fig. 3 shows that the circumference spared at this level is less than one-third of the circumference of the limb. The exclusion of sartorius would reduce the cross-section of the CTV and increase the size of the normal tissue corridor significantly. Specific measurement of the circumference left unirradiated showed that the minimum corridor was 6% of the circumference of the thigh if sartorius was included, that is a mere 2.5 cm, whilst with sartorius excluded the minimum corridor was actually 27% of the circumference (Table 3). With the exclusion of sartorius from the CTV, the size of the unirradiated corridor therefore approaches the recommendation to spare approximately a third of the circumference of the limb.[13]

### *Implications of our study*

This study was necessarily small and would benefit from being validated elsewhere. Nevertheless, we found convincing evidence, in the upper thigh, that sartorius is effectively protected from tumour invasion by its own fascial sheath. In the lower thigh, this sheath was evident in two-thirds of the subjects we examined. In the other two cases, although thinned, the sheath was no thinner than the intermuscular septum, which almost always prevents tumour penetration.

Following our anatomical investigation, we feel confident that sartorius muscle is likely to be protected from microscopic infiltration by its fascial layers. However, gross tumour does on rare occasions penetrate the intermuscular septa and fascia lata, so that protection from an adjacent tumour mass cannot be guaranteed. Full assessment, especially with MR imaging is obviously required in every case. We suggest that sartorius need not be included in the radiotherapy CTV for anterior compartment soft tissue sarcomas, provided there is no abnormality seen on pre-operative MRI. This has now become standard practice in our unit.

Similar considerations might also apply to surgical management, considered on an individual basis, but this study was designed only to address the specific radiotherapy issue of the extent of the CTV.

### References

1. *Gray's Anatomy*. 38th edn. Edinburgh: Livingston, 1995.
2. Last RJ. *Anatomy*: *Regional and Applied*. 6th edn. Edinburgh: Churchill Livingston, 1978.
3. Draves DJ. *Anatomy of the Lower Extremity*. Baltimore, MD: Williams & Wilkins, 1986.
4. Wood Jones F. *Buchanan's Manual of Anatomy*. Baillière Tindall and Cox, 1949.
5. Woodbourne R, Burkel W. *Essentials of Human Anatomy*. London: Oxford University Press, 1994.
6. Rosse C, Gaddum-Rosse P. *Hollinshead's Textbook of Anatomy*. New York: Lippinott-Raven, 1997.
7. Hall-Craggs E. *Anatomy as a Basis for Clinical Medicine*. Baltimore, MD: Urban & Schwarzenburg, 1990.
8. Suit H. The Gray Lecture 2001: coming technical advances in radiation oncology. *Int J Radiat Oncol Biol Phys* 2002; 53: 798–809.
9. Stinson SF, DeLaney TF, Greenberg J, Yang JC, Lampert MH, Hicks JE, Venzon D, White DE, Rosenberg SA, Glatstein EJ. Acute and long-term effects on limb function of combined modality limb sparing therapy for extremity soft tissue sarcoma. *Int J Radiat Oncol Biol Phys* 1991; 21: 1493–9.
10. Robinson MH, Bidmead AM, Harmer CL. Value of conformal planning in the radiotherapy of soft tissue sarcoma. *Clin Oncol* 1992; 4: 290–3.
11. Robinson MH. Post-treatment limb function in soft tissue sarcomas. In: Verweij J, Pinedo HM, Suit HD, eds. *Soft Tissue Sarcomas*: *present achievements and future prospects*. Boston, MA: Kluwer Academic Publishers, 1997.
12. Karasek K, Constine LS, Rosier R. Sarcoma therapy: functional outcome and relationship to treatment parameters. *Int J Radiat Oncol Biol Phys* 1992; 24: 651–6.
13. Swedish Council on Technology Assessment in Health Care (SBU). Radiotherapy for Cancer. *Acta Oncol* 1996; 35 (Suppl 6: 1–100; Suppl 7: 1–152).
